# Hypodermic Medication

**Published:** 1907-05-25

**Authors:** 


### RESIDENT MEDICAL OFFICERS' DEPARTMENT.

### HYPODERMIC MEDICATION.

THE modern irrational custom of giving routine hypodermic injections in haphazard fashion for shock and pain is greatly to be deplored. At the proper time and under certain conditions subcutaneous injections are immensely superior to all other methods of treatment. But the dangers attending loose and unconsidered injections of potent drugs cannot be too strongly insisted upon. Furthermore, one thoughtless hypodermic injection, never repeated by the physician, may become the starting-point of an irrevocable habit in the patient. Accurate dosage and certain and immediate effects are great advantages, and no injection should ever be given without careful consideration of all the circumstances of the case.

Medical students do not always receive sufficient practical instruction in the administration of hypodermic injections. They are seldom in the wards at the times when the house officer is called upon to apply this form of treatment. The result is that many newly qualified practitioners enter upon house appointments or commence private practice with imperfect knowledge of the principles of hypodermic medication. In the first place, we recommend the junior practitioner to have his own hypodermic outfit and to keep it always at hand. Emergencies in which the prompt injection of strychnine, morphine, or apomorphine means life or death to the patient arise at the most unlikely times, and even in hospital it is as well not to trust to others for the right things being in the right place at the right moment. A good all-glass aseptic syringe with three or four tubes of compressed drugs can nowadays be carried in the most convenient form, and it can be relied upon to act efficiently at the shortest notice. With his own outfit in his waistcoat pocket, the house surgeon is prepared for emergencies and is independent alike of excited nurses, of impervious needles, and of adherent phial stoppers.

With regard to the administration of hypodermic injections in the wards by nurses, the resident medical officer must be guided by the rules of his own hospital and, of course, by his own judgment. In some hospitals the night nurses are allowed, under certain restrictions, to give injections when expressly ordered by the resident; in others, only the sister of the ward and the night sister are permitted to do so. The former plan, in spite of certain obvious objections, is, in our opinion, the better, for there are cases in which the inevitable delay in sending for the sister or house officer to administer the prescribed injection may prove fatal to the patient.

The resident, when he anticipates the occurrence for strychnine or morphine, should write a prescription for this purpose, and should give explicit directions as to the circumstances under which it is to be used and as to whether he himself is also to be summoned to the case. Once given, the hypodermic should not be repeated without his express sanction; and his instructions should be renewed from day to day, so long as the patient's state renders the necessity for a prompt injection probable or even possible.

Usually every ward is supplied with a syringe and small bottles of the B.P. injections of morphine and of strychnine. The house surgeon should periodically test the efficiency of the syringe, while the injections should at intervals be replaced by freshly prepared solutions. The china tray for this apparatus should also contain bottles of distilled water and of ether or alcohol, together with two small beakers and a supply of swabs. After each injection the needle should be boiled with the wire in its lumen, and, of course, a separate outfit should be kept for typhoid or other infectious cases. Running first alcohol and then air through the needle prevents rusting and prolongs its life.

As for the injection itself, it is hardly necessary to say more than a word or two. Few medical men, however, are as careful about cleaning up the site of the puncture as they should be. An elaborate preparatory dressing is unnecessary, but a few passes over the skin with a swab of alcohol take no time and form a rational precaution. If the resident happens to inject morphia into a vein instead of into the subcutaneous tissues—a very rare occurrence even in the dark—both he and his patient will have a very rude shock. To avoid this catastrophe one may detach the body of the charged syringe from the needle after the latter has been thrust under the skin, and observe whether blood flows in any quantity from the lumen of the needle. This is quite easy with an all-glass syringe, and occupies only a moment or two. It is only necessary when the veins cannot be seen through the skin, and when deep intra-muscular injections of mercury or ergotin are administered.

A word of warning may be given on the danger of repeated injections of strychnine to collapsed or apparently moribund patients. Bold and continued doses have resulted before now in the patient emerging from one precarious condition only to subside into a state of strychnine poisoning, recognised when it is too late and impossible to overcome. This is especially true in the case of children, who often tolerate strychnine almost as badly as they do opium. Finally, we would remind the junior practitioner, whether in hospital or in private practice, that there are various efficient substitutes for morphia in selected cases. As we have said above, subcutaneous injection should only be given when absolutely necessary. Pil. saponis co. is an excellent method of giving opium when it is advisable to conceal the nature of the treatment from the patient or the attendants; and we might add that an injection of sterile water has often produced sleep and relieved imaginary pains in neurotic or over-anxious patients. The resident should also remember that, when a fair dose of morphia fails in its effect, it may be that he has to deal with the victim of a drug habit.

